# Investigation of factors affecting annual milk yield of different goat breeds using panel data analysis

**DOI:** 10.1371/journal.pone.0341421

**Published:** 2026-01-27

**Authors:** Esra Yavuz

**Affiliations:** Şırnak University, Cizre Vocational School, Department of Accounting and Tax, Cizre, Şırnak, Turkey; Eskisehir Osmangazi University: Eskisehir Osmangazi Universitesi, TÜRKIYE

## Abstract

This study aims to evaluate the effects of basic biological factors such as age and lactation length on milk yield by examining the annual milk yield data of five different goat breeds (Damaskus, Pure Damaskus, Toros, Çukurova, and German Fawn) raised in Turkey between 1991 and 1997. The study used a panel data analysis method that takes into account both horizontal (between races) and vertical (within time) variability. It includes annual milk yield (liters), lactation period (days), and age (years) variables of randomly selected individuals from each race. Fixed effects and random effects models were compared in the statistical analysis, and the fixed effects model was preferred according to the Hausman test results. The findings revealed that both age and lactation period had positive and statistically significant effects on milk yield. In addition, significant differences were found among the breeds in terms of milk yield; it was observed that the Çukurova breed had higher performance, while the Toros breed had lower milk yield. In conclusion, this study shows that breed selection and production management play a critical role in productivity in goat breeding and reveals that panel data analysis can be used as an effective method in animal production research.

## Introductıon

Goat farming is an important source of livestock in terms of milk, meat, and fiber production for people in the world. Goats are known as the most preferred animal husbandry due to their high adaptability to environmental conditions, low maintenance requirements, and ability to adapt to different geographical regions. In terms of these features, goat farming makes an important contribution to sustainable agricultural practices [1,2].

Dairy goat farming is an important source of income, especially in small animal farms. However, there are many biological and environmental factors that affect milk yield. The most important of these are breed differences, age of the animal, and lactation period. While the genetic potential of different goat breeds is a determinant of milk yield, physiological development and production capacity also change with age. In addition, the lactation period is an important parameter affecting both milk yield and milk quality [3,4].

The panel data analysis is an important econometric method preferred in the analysis of agricultural production data as it provides more observations and densities by taking into account both time and unit dimensions [5,6]. Since this method offers the opportunity to control the heterogeneity between variables, it allows for more accurate estimation of the effects on production performance of different goat breeds. In the analyses performed, fixed effects and random effects models were compared; the appropriate model was examined with the Hausman test. Thus, the application of the Panel data structure revealed the effects of factors that change over time and differ between breeds in more detail. It is aimed that the findings obtained will provide a scientific basis for applications such as breed and age selection to increase productivity in goat breeding. This study aims to comparatively evaluate the performance of five different goat breeds, Damascus hybrid, Pure Damascus, Toros, Çukurova, and German Fawn goats, which are widely used in milk production, in terms of milk yield, lactation period, and age. The panel data analysis method was used to evaluate the data between variables.

Additionally, to achieve sustainable productivity increases in goat farming, it is crucial to analyze the biological factors affecting production performance using scientific methods. Accurately identifying variability in milk yield among different breeds plays a critical role in both guiding producers’ breed preferences and in interregional production planning. In this context, evaluating long-term datasets containing multiple breeds using econometric methods offers the opportunity to more objectively examine the effects of both genetic potential and physiological traits on production. Therefore, this study aims to contribute to the scientific knowledge that can improve decision-making processes in goat farming.

## Materials and methods

This study was conducted with data including milk yield, lactation period, and age information of five different goat breeds raised in Turkey between 1991 and 1997. The goat breeds examined were Damascus hybrid, Pure Damascus, Toros, Çukurova, and German Fawn, and were selected to represent both local and culture breeds. Annual milk yield (MEF) (liter), lactation period (LT) (day), and age (AGE) (year) variables of randomly selected individuals from each breed were recorded regularly between 1991–1997.

### Dataset and variables

The data used in the study included the following variables on an annual basis for five different goat breeds between 1991 and 1997:

- Annual Milk Yield (liters) – Dependent variable
- Age (years) – Independent variable
- Lactation Length (days) – Independent variable
- Breed – Panel cross-section unit
- Year (1991–1997) – Time dimension

The research model is based on panel data analysis, which has both cross-sectional (breeds) and time (1991–1997) dimensions and allows analyzing how variables differ over time. The panel data method produces more accurate and consistent results on agricultural and animal production data, thanks to the ability to control for fixed differences between individuals.

In the study, pearson correlation coefficients between the determined characteristics were determined first. Variables were standardized by regression analysis, with real milk yield being dependent and other variables being independent. Panel data analysis was applied in the analysis of data using Eviews 9.

In the study, firstly, Spearman correlation analysis was performed to determine whether there is a multicollinearity problem among the independent variables. Then, the dependency between the cross-sections forming the panel was examined with [7] (Lagrange Multiplier-LM) and [8] (Cross-Section Dependence-CD) tests on the basis of panel and variable. The variability of the coefficients of the variables between the cross-sections was investigated with the Homogeneity Test developed by [9]. Homogeneity test was examined separately according to whether the constant terms α and β (effect on the dependent variable) of each variable (their effect on the dependent variable) were homogeneous or heterogeneous on a variable basis and excluding explanatory variables. Stationarity of the series was analyzed with the second generation unit root test of [10] PANIC, which takes into account cross-sectional dependency, and the first generation tests of Im, [11] IPS and Levin, [12] LLC, which takes into account homogeneity/heterogeneity. In order to choose which method to use to estimate the model, the F test, [7,13,14] were applied. The heteroscedasticity, which expresses the situation that the error term variance in the models is not the same for all observations, was examined with Breusch-Pagan-Godfrey Heteroscedasticity LM. Model estimation was carried out using the Period SUR (PCSE) method, which corrects panel standard errors developed by [15].

### Investigation of the multiple linearity problem

A complete or nearly complete linear relationship between all or some of the independent variables in the panel regression model is called multiple linearity. A high degree of correlation between independent variables can make it impossible to calculate the parameters and render the least squares method unusable. Therefore, in panel data analysis, firstly, whether there is a multicollinearity problem among the independent variables was investigated with Spearman correlation analysis and variance inflation test which are frequently used in the literature. Variables with values above the critical values that may cause a multicollinearity problem were removed from the analysis [16,17].

### Testing cross-section dependency

If there is a cross-sectional dependency between the series, this situation should be taken into consideration and the analysis should be carried out, which affects the accuracy and reliability of the findings [7,8]. Analysis results that do not take cross-section dependency into account may become biased and inconsistent. In this direction, it is necessary to test whether there is cross-sectional dependency in the series before panel data analysis. The existence of cross-sectional dependency between the series can be determined by [7] LM test, [8] CD and CDlm tests or [18] LMadj test. [7] LM test is used when the time dimension is much larger than the cross-section dimension (T > N), [8] CDlm test is used when the time dimension is larger than the cross-sectional dimension (T > N), but the difference between the two dimensions is not large. [8] CD test is used when the cross-section dimension is larger than the time dimension (N > T), [18] LMadj test eliminates the deviations in the LM test and the possibility of the total correlation in the Pesaran CD test being 0 and is used when the T dimension is larger than the N dimension.

The existence of cross-sectional dependency among the units forming the panel was examined separately on a panel and variable basis with the help of Gaussian codes.

### Panel unit root tests

In order to perform panel data analysis and obtain accurate results, the stationarity of the time series of the variables must be ensured [16,19]. In other words, in order to obtain meaningful results between dependent and independent variables, the series must be stationary. In panel data analysis, it is necessary to determine whether the cross-sections are independent of each other before the unit root test. At this point, panel unit root tests are divided into two groups as first and second generation tests in line with their consideration of cross-sectional dependency [20,21]. First-generation unit root tests are based on the assumption that cross-sectional units are independent of each other and that a shock occurring in one of the sections forming the panel affects all sections at the same level. In addition, these tests are divided into two groups as homogeneous and heterogeneous models. While the [12,13] tests are based on the assumption of homogeneity; the [11,22,23] tests are based on the assumption of heterogeneity. The [24] test is based on both the assumption of homogeneity and heterogeneity. The second generation unit root tests are based on the assumption that the cross-section units are not independent from each other and that the shock occurring in one of the cross-sections forming the panel affects all cross-sections at different levels. In this context, the second generation unit root tests were developed that take into account the cross-sectional dependence between the cross-sections [25].

### Estimation of panel data models

Firstly, Fixed Effects (FE) and Random Effects (RE) models were estimated. The Hausman test was applied to choose between the two models. Hausman test results showed that the fixed effects model provided more reliable and consistent estimates. Therefore, the fixed effects model was preferred in the analyses [20,26].

Before moving on to panel regression analysis, the model should be tested for deviations from the assumptions of multiple regression analysis. The assumptions of multiple linear regression are:

- Multicollinearity
- Heteroscedasticity
- Autocorrelation

The test results of the assumptions obtained from the Eviews 9 program are given in equations (1), (2), (3), (4), and (5) below, separately for each panel data model.

### Model Estimation Results


$$\text{ln}{(\text{LnGERMEF}}_{\text{it}})=\beta_{\text{0}}+\beta_{\text{1}}\text{ln}\left({\text{LnGERLT}}_{\text{it}}\right)+\beta_{\text{2}}\text{ln}\left({\text{LnGERAGE}}_{\text{it}}\right)+\mu_{\text{it}}$$ (1)

$$\text{ln}{(\text{LnCKVMEF}}_{\text{it}})=\beta_{\text{0}}+\beta_{\text{1}}\text{ln}\left({\text{LnCKVLT}}_{\text{it}}\right)+\beta_{\text{2}}\text{ln}\left({\text{LnCKVAGE}}_{\text{it}}\right)+\mu_{\text{it}}$$ (2)

$$\text{ln}{(\text{LnDMSMEF}}_{\text{it}})=\beta_{\text{0}}+\beta_{\text{1}}\text{ln}\left({\text{LnDMSLT}}_{\text{it}}\right)+\beta_{\text{2}}\text{ln}\left({\text{LnDMSAGE}}_{\text{it}}\right)+\mu_{\text{it}}$$ (3)

$$\text{ln}{(\text{LnPDMSMEF}}_{\text{it}})=\beta_{\text{0}}+\beta_{\text{1}}\text{ln}\left({\text{LnPDMSLT}}_{\text{it}}\right)+\beta_{\text{2}}\text{ln}\left({\text{LnPDMSAGE}}_{\text{it}}\right)+\mu_{\text{it}}$$ (4)

$$\text{ln}{(\text{LnTRSMEF}}_{\text{it}})=\beta_{\text{0}}+\beta_{\text{1}}\text{ln}\left({\text{LnTRSLT}}_{\text{it}}\right)+\beta_{\text{2}}\text{ln}\left({\text{LnTRSAGE}}_{\text{it}}\right)+\mu_{\text{it}}$$ (5)

In the equations, i represents the cross-sectional units, t represents the time dimension, $\beta_{\text{0}}$ represents the constant variable, $\beta_{\text{n}}$ represents the slope coefficient of the nth variable, and μ represents the error term. In addition, the logarithmic expressions in the equations used in the study indicate that the natural logarithms of the dependent and independent variables are included in the model.

## Findings and discussion

When the descriptive statistics results are evaluated in Table 1, the average value of the German Fawn breed is higher than the other breeds. Similarly, the average value of the age variable is higher than the other breeds. Thus, the German Fawn breed has the highest milk yield and long lactation period, making it an ideal breed for milk production. In addition, it was observed that the average milk yield difference between the German Fawn breed and other breeds is high. This shows that it depends on genetic differences and environmental conditions. When the skewness value is examined, it is observed that the value is negative in all breeds and takes values close to zero. In other words, it shows that the distribution is skewed to the left.

**Table 1:** Descriptive statistics.

	German Fawn			Çukurova			Damascus			Pure Damascus			Toros		
Stats.	MEF	LT	AGE	MEF	LT	AGE	MEF	LT	AGE	MEF	LT	AGE	MEF	LT	AGE
Mean	242.38	200.66	5.66	5.48	5.33	1.65	5.50	5.32	1.65	5.37	5.29	1.65	5.531	5.34	1.65
Median	238.50	210	6	5.52	5.37	1.79	5.55	5.38	1.79	5.42	5.30	1.79	5.54	5.38	1.79
Max.	583	263	9	6.03	5.60	2.19	6.22	5.70	2.19	5.99	5.55	2.19	6.05	5.57	2.19
Min.	100	114	2	4.69	4.66	0.69	4.64	4.70	0.69	4.62	4.73	0.69	4.61	4.69	0.69
Std. Dev.	81.69	36.37	2.06	0.26	0.16	0.41	0.36	0.19	0.41	0.34	0.17	0.41	0.28	0.17	0.41
Skewness	0.69	−0.79	−0.00	−0.43	−1.31	−0.60	−0.49	−1.27	−0.60	−0.40	−1.08	−0.60	−0.60	−1.72	−0.60
Kurtosis	4.07	2.93	1.87	3.09	5.10	2.48	2.70	4.21	2.48	2.31	4.12	2.48	3.26	6.07	2.48
Jarque-Bera	16.12	13.18	6.65	4.03	59.65	9.05	5.59	41.87	9.05	5.80	31.45	9.05	7.96	112.17	9.05
Probability	0.00	0.001	0.03	0.13	0.00	0.01	0.06	0.000	0.01	0.05	0.00	0.01	0.01	0.00	0.01
Sum	30541.0	25284	714	690.7	671.8	208.9	693.3	671.5	208.9	676.7	666.7	208.9	696.9	673.4	208.9
Obs.	126	126	126	126	126	126	126	126	126	126	126	126	126	126	126

In Table 2, the correlation coefficients between the independent variables were examined to test the assumption of multicollinearity, which indicates that there is no complete relationship between the independent variables. The problem of multicollinearity is the situation where there is a high degree of correlation between the variables. A correlation coefficient above 0.90 between the variables creates a problem. When Table 2 is examined, the highest correlation coefficient between the lactation period and age variables was calculated as 0.61. This value is not critical in terms of multicollinearity. Thus, it indicates that there is no multicollinearity problem between the variables.

**Table 2:** Spearman correlation matrix for multicollinearity.

Correlation German Fawn	MEF	LT	AGE
MEF	1.00		
LT	0.61^***^	1.00	
AGE	0.08	−0.11	1.00
Correlation Çukurova	MEF	LT	AGE
MEF	1.00		
LT	0.51^***^	1.00	
AGE	0.08	0.01	1.00
Correlation Damascus	MEF	LT	AGE
MEF	1.00		
LT	0.60^***^	1.00	
AGE	−0.08	−0.12	1.00
Correlation Pure Damascus	MEF	LT	AGE
MEF	1.00		
LT	0.49^***^	1.00	
AGE	−0.15^*^	−0.13	1.00
Correlation Toros	MEF	LT	AGE
MEF	1.00		
LT	0.52^***^	1.00	
AGE	0.02	−0.06	1.00

Endogeneity is the situation where one of the independent variables in regression analysis is related to the error term. When Table 3 is examined, it is observed that the probability of being related to the error term between milk yield and age variables in 5 different breeds is low. In addition, the probability of being related to the error term among other variables is low.

**Table 3:** Spearman correlation matrix for endogeneity.

Correlation German Fawn	ERRORTERM	LT	AGE
ERRORTERM	1.00		
LT	0.02	1.00	
AGE	0.03	−0.11	1.00
Correlation Çukurova	ERRORTERM	LT	AGE
ERRORTERM	1.00		
LT	0.02	1.00	
AGE	−0.05	0.01	1.00
Correlation Damascus	ERRORTERM	LT	AGE
ERRORTERM	1.00		
LT	0.02	1.00	
AGE	−0.03	−0.12	1.00
Correlation Pure Damascus	ERRORTERM	MEF	MEF
ERRORTERM	1.00		
LT	0.01	1.00	
AGE	−0.02	−0.13	1.00
Correlation Toros	ERRORTERM	LT	AGE
ERRORTERM	1.00		
LT	0.00	1.00	
AGE	0.00	−0.06	1.00

The existence of cross-sectional dependency among the units forming the panel was examined by performing panel analysis with Gaussian codes. The cross-sectional dependency results are given in Table 4. When the cross-sectional dependency tests performed on a panel basis are examined in Table 4, it is seen that the probability value is greater than 0.05 in all tests applied for the annual milk yield variables of Çukurova, Damascus and Toros breeds, while the probability value of all other variables is less than 0.05. Since the cross-sectional dimension is larger than the time dimension, Pesaran CD test results were taken into consideration. As a result of the analysis, since the probability values of the annual milk yield variables belonging to the Çukurova, Damascus and Toros breeds are greater than 0.05, which is accepted as the critical value, the null hypothesis cannot be rejected. On the other hand, since the probability values of the other variables are less than 0.05, the null hypothesis is rejected. While performing the stationarity test for the annual milk yield variables belonging to the Çukurova, Damascus and Toros breeds, there is no cross-sectional dependence. Thus, while performing the stationarity test for these variables, second generation unit root tests that take into account the cross-sectional dependence were applied.

**Table 4:** Results of cross-sectional dependence tests.

Variables	Pesaran CD	
	Statistic	p-value
LnGERMEF	2.92^***^	0.00
LnGERLT	5.18^***^	0.00
LnGERAGE	32.71^***^	0.00
LnCKVMEF	0.86	0.38
LnCKVLT	3.97^***^	0.00
LnCKVAGE	32.71^***^	0.00
LnDMSMEF	1.35	0.17
LnDMSLT	7.01^***^	0.00
LnDMSAGE	32.71^***^	0.00
LnPDMSMEF	6.81^***^	0.00
LnPDMSLT	5.24^***^	0.00
LnPDMSAGE	32.71^***^	0.00
LnTRSMEF	1.48	0.13
LnTRSLT	8.82^***^	0.00
LnTRSAGE	32.71^***^	0.00

H0: There is no dependency between sections

H1: There is a dependency between the sections.

If there is no cross-sectional dependence between the series in panel data analysis, heterogeneity tests should be applied to determine the unit root tests that should be applied for stationarity [27].

Table 5 shows the analysis results of the slope heterogeneity test for five different races and for each variable separately.

**Table 5:** Results of slope heterogeneity tests.

Variables	$\tilde{\Delta }$	P-value	${\tilde{\Delta }}_{adj}$	P-value
LnGERMEF	−0.38	0.65	−0.50	0.69
LnGERLT	−0.08	0.53	−0.11	0.54
LnGERAGE	1.85^**^	0.03	2.45^***^	0.00
LnCKVMEF	−0.87	0.80	−1.15	0.87
LnCKVLT	−0.74	0.77	−0.98	0.83
LnCKVAGE	1.85	0.03	2.45	0.00
LnDMSMEF	−1.71	0.95	−2.27	0.98
LnDMSLT	−1.31	0.90	−1.74	0.95
LnDMSAGE	1.85	0.03	2.45	0.00
LnPDMSMEF	−1.51	0.93	−2.00	0.97
LnPDMSLT	−0.24	0.59	−0.32	0.62
LnPDMSAGE	1.85	0.03	2.45	0.00
LnTRSMEF	0.11	0.45	0.15	0.44
LnTRSLT	−1.81	0.96	−2.39	0.99
LnTRSAGE	1.85	0.03	2.45	0.00

$\tilde{\Delta }:$Delta Tilde, ${\tilde{\Delta }}_{adj}:$ Corrected Delta Tilde

H0: There is heterogeneity.

H1: There is no heterogeneity.

As a result of the heterogeneity analysis, it was determined that the probability values of the age variables for five different breeds were less than the critical value of 0.05. Therefore, the null hypothesis is rejected. In other words, it was determined that these variables were homogeneous. The probability values of the lactation period and annual milk yield variables for five different breeds were above the critical value and the null hypothesis was not rejected. In other words, these variables were heterogeneous. Stationarity tests, first generation unit root tests that do not take into account cross-sectional dependence and have a heterogeneous structure were applied for lactation period and annual milk yield variables for five different breeds. In addition, tests with a homogeneous structure were applied for age variables for five different breeds.

The stationarity of the variables used in the study was determined by taking into account the cross-sectional dependency and homogeneity conditions. As a result of the cross-sectional dependency test, it was determined that cross-sectional dependency was detected between the lactation period, annual milk yield and age variables of five different breeds. Pesaran CIPS (Cross-Sectionally Augmented IPS) test was used for stationarity tests of the variables. Pesaran CIPS test results are shown in Table 6.

**Table 6:** Results of panel 2nd generation unit root tests.

Pesaran CIPS				
	Intercept		Intercept and trend	
Variables	CIPS t-stat.	p-value	CIPS t-stat.	p-value
LnGERMEF	−2.84^***^	<0.01	−4.38^***^	<0.01
LnGERLT	−2.57^**^	<0.05	−3.10^**^	<0.05
LnGERAGE	−6.19^***^	<0.01	−6.42^***^	<0.01
LnCKVLT	2.40^**^	<0.05	−4.24^***^	<0.01
LnCKVAGE	−6.19^***^	<0.01	−6.42^***^	<0.01
LnDMSLT	−2.67^**^	<0.05	−4.10^***^	<0.01
LnDMSAGE	−6.19^***^	<0.01	−6.42^***^	<0.01
LnPDMSMEF	−2.39^**^	<0.05	−4.51^***^	<0.01
LnPDMSLT	−2.31^*^	<0.10	−3.08^**^	<0.05
LnPDMSAGE	−6.19^***^	<0.01	−6.42^***^	<0.01
LnTRSLT	6.50^***^	<0.01	−8.85^***^	<0.01
LnTRSAGE	−6.19^***^	<0.01	−6.42^***^	<0.01
Critical values	1%	−2.69	1%	−3.52
	5%	−2.36	5%	−3.06
	10%	−2.20	10%	−2.85

The values of the test statistics obtained as a result of the analysis in Table 6 were compared with the Pesaran CIPS critical values to test stationarity for each variable. Since the CIPS critical table value was greater than the CIPS test statistic value obtained, the null hypothesis ($H_{\text{0}}:$ The series has a unit root) was rejected, and it was concluded that the series was stationary.

For the stationarity of the annual milk yield variables belonging to the Çukurova, Damascus, and Toros breeds, which were determined to have no cross-sectional dependence but to have a heterogeneous structure as a result of the homogeneity test, the Levin, Lin & Chu (LLC) test, which is one of the first generation root tests on Panel and has a heterogeneous structure, was applied. The results of the first-generation unit root tests on the annual milk yield variables for the Çukurova, Damascus, and Toros breeds are presented in Table 7.

**Table 7:** Results of panel 1nd generation unit root tests.

Levin, Lin & Chu (LLC)	Intercept		Intercept and trend	
Variables	LLC t-stat.	p-value	LLC t-stat.	p-value
LnCKVMEF	−10.71	0.00	−14.50	0.00
LnDMSMEF	−9.43	0.00	−14.39	0.00
LnTRSMEF	−13.2	0.00	−18.893	0.00

### Panel regression analysis results

In the panel regression analysis, an attempt was made to determine whether there is a statistically significant relationship between the dependent and independent variables and if so, the direction of this relationship. As a result of the analysis, it was determined that the variances of the error terms in the model to be estimated were not constant and the successive values of the error terms were not independent of each other. Thus, in this study, the Period SUR method developed by [15] addressing these problems was used to correct the panel standard errors and the estimation results are given in Table 8.

**Table 8:** Estimation of models with Driscoll-Kray estimator.

Race	German Fawn		Cukurova		Damascus		Pure Damascus		Toros	
	Panel A		Panel B		Panel C		Panel D		Panel E	
Regressors	Coeff.	p-value.	Coeff.	p-value.	Coeff.	p-value.	Coeff.	p-value.	Coeff.	p-value.
LNSURE	1.16^***^	0.00	0.89	0.00	1.51	0.00	0.89^***^	0.00	1.02	0.00
LNYAS	0.10^**^	0.01	0.45	0.15	0.66	0.07	−0.97^**^	0.03	0.38	0.00
C	−0.87	0.25	−0.05	0.94	−3.67	0.00	2.24^**^	0.01	−0.59	0.21
Adj.R^2^	0.454		0.39		0.64		0.55		0.42	
F-stat	53.00^***^		4.32^***^		10.17^***^		7.17^***^		12.39^***^	
J-B stat	0.42		3.09		1.03		0.82		3.06	
LR stat	18.61		10.75^***^		10.69^***^		30.57^***^		15.48	
LMp-stat	1.35		2.46		0.47		1.95		5.76^**^	
CSD stat	−0.22		−1.86^*^		−1.82^*^		−1.70^*^		−1.83^*^	
F-group	0.80		1.86^**^		2.07^**^		3.17^***^		0.98	
F-time	0.71		2.49^**^		6.89^***^		7.28^***^		3.33^***^	
F-twoway	0.79		2.01^***^		3.33^***^		4.29^***^		1.81^**^	
Honda-group	−0.59		1.60^*^		1.24		2.72^***^		0.17	
Honda-time	−0.76		1.41^*^		5.06^***^		5.48^***^		2.73^***^	
Honda-twoway	−0.96		2.13^**^		4.45^***^		5.80^***^		2.05^**^	

Table 8 shows the model estimated values with the Driscoll-Kray estimator. As a result of these values, the estimation results performed in line with the model created to determine the variables affecting lactation yield are given. When the results are examined, the F statistic probability values expressing the significance of the model as a whole are significant for the regression models and the Adj. $R^{\text{2}}$ values are obtained as 0.45, 0.39, 0.64, 0.55, and 0.42, respectively. In addition, the Honda test result was significant in the lactation yield of Çukurova, Damascus, Pure Damascus and Toros breeds. In other words, it can be said that there is a structural difference in the lactation yield of the breeds.

## Conclusıon

This study aims to analyze the milk yield, lactation period, and age variables of five different goat breeds raised in Turkey between 1991 and 1997 using the panel data method. The purpose of choosing panel data analysis in the study is to present more accurate results with data that include both time and breed variability.

Fixed effects model was applied in the analysis and the Hausman test results showed that this model should be preferred. Fixed effects model allows isolating the effect of fixed effects of each breed on milk yield over time.

According to the findings, it was determined that the age, variable had a statistically significant effect on milk yield. This situation shows that milk yield increases with increasing age of goats. However, this increase is valid up to a certain age and it should be kept in mind that there may be a decrease in yield at later ages. Similarly, it was observed that the extension of the lactation period significantly increases milk yield. This result reveals that longer lactation periods contribute to more milk production.

A general increasing trend in milk yield was detected from 1991 to 1997. This increase may be the result of improvements in feeding conditions, management practices, or genetic selection studies.

In the comparisons made between the breeds, it was seen that the Cukurova breed had the highest fixed effect in terms of annual milk yield; this shows that the breed in question may have a genetically superior milk production capacity. On the other hand, the milk yield of the Pure Damascus breed was lower than the other breeds and had the lowest fixed effect.

The coefficient sizes of variables such as age and lactation length, which influence milk yield, in the model more clearly demonstrate the impact of these factors on production. The higher elasticity coefficient for lactation length compared to age suggests that the physiological dynamics of lactation are more decisive in milk production. Conversely, the more limited but steady increase in the effect of age suggests that goats reach their highest milk yield at a specific maturity stage and that yield may decline at later ages due to regression in mammary tissue. The significant differences in fixed effects between breeds highlight the critical role of genetic capacity in milk yield, particularly demonstrating that the Cukurova goat has a higher milk potential compared to other breeds.

The findings of this study are consistent with similar studies in the literature. For example, in a study conducted by [27], milk yield and components of Saanen and German Fawn goat crossbreeds were examined and it was stated that both breeds showed high milk yield. In addition, in a study conducted by [28], it was stated that the Çukurova breed was prominent in terms of milk yield compared to other breeds. In addition, in a study conducted by [29], the behaviors of dairy goat breeds in hot and cold climate conditions in Çukurova conditions were examined and it was stated that these breeds had high adaptation abilities. Thus, it can be evaluated as a reason why the Çukurova breed is more advantageous in terms of milk yield. [30] compared the milk yield and milk components of Damascus (Shami) goats and German Fawn × Hair crossbred goats under Mediterranean climate conditions. In this study, both genotypes were reported to have high milk yields and similar milk composition. This result supports our finding of differences in milk yield performance between the breeds in our study.

As a result of these findings, they provide important clues for researchers and breeders who want to increase milk production. Choosing the right breed, especially in terms of milk yield, is very important as it will increase production efficiency. In addition, careful planning of the lactation period and keeping the animals in the production age range will further increase the yield. As a result, it has been revealed that managerial practices are as much a determinant in milk production as genetic factors.

## Supporting information

S1 Data (XLSX)

## References

[1] Gök Y, Şahin M, Yavuz E. Comparatıve analysıs of indıvıdual lactatıon curve models in some cattle breeds. 2021.

[2] Tirink C, Önder H, Yurtseven S, Akil ZK. Comparison of some non-linear functions to describe the growth for Linda geese with CART and XGBoost algorithms. Czech J Anim Sci. 2022;67(11):454–64. doi: 10.17221/129/2022-cjas

[3] Ağyar O, Tırınk C, Önder H, Şen U, Piwczyński D, Yavuz E. Use of Multivariate Adaptive Regression Splines Algorithm to Predict Body Weight from Body Measurements of Anatolian buffaloes in Türkiye. Animals (Basel). 2022;12(21):2923. doi: 10.3390/ani12212923 36359047 PMC9657142

[4] Sen U, Onder H. The effect of estrus synchronization programmes on parturition time and some reproductive characteristics of Saanen goats. Journal of Applied Animal Research. 2015;44(1):376–9. doi: 10.1080/09712119.2015.1091348

[5] Abaci SH, Onder H, Sahin M, Yavuz E. Determination of Number and Position of Knots in Cubic Spline Regression for Modeling Individual Lactation Curves in Three Different Breed. PJZ. 2020;52(6). doi: 10.17582/journal.pjz/20200129080117

[6] Onder H, Tırınk C. Bibliometric analysis for genomic selection studies in animal science. Journal of the Institute of Science and Technology. 2022;12(3):1849–56.

[7] Breusch TS, Pagan AR. The Lagrange Multiplier Test and its Applications to Model Specification in Econometrics. The Review of Economic Studies. 1980;47(1):239. doi: 10.2307/2297111

[8] Pesaran MH. General diagnostic tests for cross section dependence in panels. Cambridge Working Papers. 2004;1240(1):1.

[9] Hashem Pesaran M, Yamagata T. Testing slope homogeneity in large panels. Journal of Econometrics. 2008;142(1):50–93. doi: 10.1016/j.jeconom.2007.05.010

[10] Bai J, Ng S. A PANIC Attack on Unit Roots and Cointegration. Econometrica. 2004;72(4):1127–77. doi: 10.1111/j.1468-0262.2004.00528.x

[11] Im KS, Pesaran MH, Shin Y. Testing for unit roots in heterogeneous panels. Journal of Econometrics. 2003;115(1):53–74. doi: 10.1016/s0304-4076(03)00092-7

[12] Levin A, Lin C-F, James Chu C-S. Unit root tests in panel data: asymptotic and finite-sample properties. Journal of Econometrics. 2002;108(1):1–24. doi: 10.1016/s0304-4076(01)00098-7

[13] Honda Y. Testing the Error Components Model with Non-Normal Disturbances. The Review of Economic Studies. 1985;52(4):681. doi: 10.2307/2297739

[14] Hausman JA. Specification tests in econometrics. Econometrica. 1978;1251–71.

[15] Beck N, Katz JN. What To Do (and Not to Do) with Time-Series Cross-Section Data. Am Polit Sci Rev. 1995;89(3):634–47. doi: 10.2307/2082979

[16] Topaloğlu EE. Determination of factors affecting financial fragility in banks using panel data analysis. Eskişehir Osmangazi University Journal of Economics and Administrative Sciences. 2018;13(1):15–38.

[17] Topaloğlu EE, Ege İ. The relationship between credit default swaps (CDS) and Borsa Istanbul 100 index: Short and long term time series analyses. Journal of Business Research. 2020;12(2):1373–93.

[18] Pesaran MH. A simple panel unit root test in the presence of cross-section dependence. J of Applied Econometrics. 2007;22(2):265–312. doi: 10.1002/jae.951

[19] Gujarati D. Basic Econometrics. New York: McGraw Hill Book Co. 2003.

[20] Topaloğlu EE. Determining firm-specific factors affecting capital structure using panel data analysis: An application on corporate governance index. Finance, Political and Economic Comments. 2018;640:763–800.

[21] Breitung J. A Parametric approach to the Estimation of Cointegration Vectors in Panel Data. Econometric Reviews. 2005;24(2):151–73. doi: 10.1081/etc-200067895

[22] Maddala GS, Wu S. A Comparative Study of Unit Root Tests with Panel Data and a New Simple Test. Oxf Bull Econ Stat. 1999;61(S1):631–52. doi: 10.1111/1468-0084.0610s1631

[23] Choi I. Unit root tests for panel data. Journal of International Money and Finance. 2001;20(2):249–72. doi: 10.1016/s0261-5606(00)00048-6

[24] Hadri K. Testing for stationarity in heterogeneous panel data. The Econometrics Journal. 2000;3(2):148–61. doi: 10.1111/1368-423x.00043

[25] Topaloglu EE, Ege I, Koycu E. Coronavirus (Covid-19) and Stock Market: Empirical Analysis with Panel Data Approach. IJEF. 2021;13(3):31. doi: 10.5539/ijef.v13n3p31

[26] Kırılmaz H, Ateş H, Ünsal A. The Role of Health Indicators in Economic Growth: A Panel Regression Analysis on Turkic Republics. Eurasian Journal of International Research. 2019;7(16):35–56.

[27] Yaman S. Impact of bank-specific factors on bank profitability: Panel data analysis on Turkish banking sector. Journal of Economic and Administrative Approaches. 2021;3(2):77–100.

[28] Keskin M, Gül S, Bayraktar M. The effects of crossbreeding hair goats with Saanen and German Fawn on milk yield and milk composition. Turkish Journal of Veterinary and Animal Sciences. 2004;28(3):553–9.

[29] Yılmaz Ö. The domestic livestock resources of Turkey: Goat. Food and Agriculture Organization of the United Nations (FAO). 1996. https://www.academia.edu/10191877

[30] Keskin M, Avşar YK, Biçer O, Güler MB. A comparative study on the milk yield and milk composition of two different goat genotypes under the climate of the Eastern Mediterranean. Turkish Journal of Veterinary & Animal Sciences. 2004;28(3):531–6.

